# Post-inflammatory polyps and risk of dysplasia in inflammatory bowel disease: Wolves in sheep’s clothing?

**DOI:** 10.1055/a-2818-9346

**Published:** 2026-03-16

**Authors:** Elena De Cristofaro, Antonio Fonsi, Giovanni Monteleone, Irene Marafini

**Affiliations:** 1 60259 Gastroenterology Unit, Policlinico Universitario Tor Vergata, Rome, Italy; 2 9318 Department of Systems Medicine, University of Rome Tor Vergata, Rome, Italy

**Keywords:** Endoscopy Lower GI Tract, Inflammatory bowel disease, Polyps / adenomas / ..., Colorectal cancer

## Abstract

Patients with longstanding and extensive inflammatory bowel disease (IBD) have an enhanced risk of colorectal cancer (CRC), which accounts for up to 10% of all IBD-related deaths. Chronicity of bowel inflammation, co-existence of primary sclerosing cholangitis, and a family history of sporadic colorectal cancer represent further risk factors for development of CRC. Colon post-inflammatory polyps are believed to be another risk factor for IBD-associated CRC, even though it remains unclear how presence of such lesions could influence CRC development. Although earlier observational studies suggested an association between post-inflammatory polyps and colorectal neoplasia, more recent studies have indicated that these lesions do not independently increase neoplasia risk. However, they may, nonetheless, complicate surveillance by obscuring dysplastic lesions, particularly when numerous, and they are best regarded as markers of chronic and recurrent mucosal inflammation. Moreover, emerging evidence suggests that a minority of post-inflammatory-like lesions may conceal or coexist with dysplasia, underscoring the diagnostic challenge posed by polypoid lesions in chronically inflamed mucosa. In this article, we review the available data about the association between post-inflammatory polyps and development of CRC in IBD and discuss how advances in technology, particularly development of artificial intelligence models integrated with endoscopy, may contribute to their appropriate management.

## Introduction

Inflammatory bowel diseases (IBD), including Crohn's disease (CD) and ulcerative colitis (UC), are chronic, inflammatory disorders affecting the gastrointestinal tract 1 and characterized by enhanced risk of local and extraintestinal complications 2 3 4 . Compared with the general population, patients with UC or Crohn's colitis are six times more likely to develop colorectal cancer (CRC), which accounts for up to 10% of all IBD-related deaths 5 .

Risk of developing CRC increases with longer duration of colitis. CRC is rarely encountered in patients who have had colitis for less than 7 years; thereafter risk increases at a rate of approximately 0.5% to 1.0% per year 6 . Therefore, current guidelines recommend regular surveillance colonoscopies to screen for colorectal neoplasia (CRN; dysplasia or CRC), typically starting 8 years after symptom onset 7 .

Large cohort studies have shown increased dysplasia detection over recent years, even though colitis-associated CRC rates appear to be decreasing in patients undergoing surveillance. This latter effect could be the result of improved medical therapies, which reduce cumulative inflammatory burden, the major driver of IBD-related colon carcinogenesis, and widespread use of maintenance therapy 8 9 10 . It is also plausible that reduced frequency of IBD-associated CRC is due to the true effect of active surveillance.

To better manage risk of IBD-associated CRC, European guidelines stratify patients with IBD into low, intermediate, or high risk for CRC, depending on several risk factors. Colitis affecting less than 50% of the colon surface area and extensive colitis with mild endoscopic or histological active inflammation are considered low-risk factors. Patients with post-inflammatory polyps, CRC in a first-degree relative older than 50 years, and extensive colitis with moderate or severe endoscopic or histological inflammation are considered at intermediate risk. Finally, a stricture within the past 5 years, dysplasia within the past 5 years in a patient who declines surgery, primary sclerosis cholangitis (PSC), and CRC in a first-degree relative younger than 50 years are considered high-risk factors. European guidelines recommend colonoscopy intervals of 5, 3, and 1 year for low-, intermediate-, and high-risk patients, respectively 7 . Endoscopy plays an increasingly central role in surveillance of patients with IBD, serving as the cornerstone for detecting dysplasia and preventing CRC. Over the past decade, technological advancements have significantly enhanced diagnostic capabilities of endoscopy. High-definition imaging, virtual and dye-based chromoendoscopy, endocytoscopy, and confocal laser endomicroscopy have all contributed to improved visualization of subtle mucosal abnormalities. More recently, integration of artificial intelligence (AI) into endoscopic practice has shown promising results in lesion detection, particularly in identifying flat and subtle dysplastic changes that are often missed by the human eye.

Although current AI systems demonstrate high accuracy in distinguishing neoplastic from non-neoplastic tissue, further refinement is needed to reliably differentiate post-inflammatory polyps from true dysplastic lesions, especially in the context of chronic inflammation.

Commonly known as pseudopolyps, post-inflammatory polyps can develop in the inflamed gut of patients with IBD 11 . They are not considered independent premalignant lesions, but instead, are regarded as markers of cumulative inflammatory burden and disease severity. Their inclusion among intermediate-risk features reflects the association between longstanding, recurrent inflammation and colorectal carcinogenesis, rather than a direct causal role of the polyps themselves.

Nonetheless, understanding the nature of post-inflammatory polyps is crucial because they contribute to the burden of surveillance colonoscopies, potentially impacting both the quality of life of patients and healthcare costs.

In this review, we discuss available data about the association between post-inflammatory polyps and development of CRC in IBD and discuss the potential role of endoscopy and AI-based systems in the appropriate assessment of these lesions.

## Post-inflammatory polyps and modulation of CRC risk in IBD

Post-inflammatory polyps are very common in IBD, especially in UC patients, and are usually located in the colon, especially the transverse colon and descending/sigmoid colon. Ileal location is more common in CD or UC with backwash ileitis 12 . Prevalence is similar in both sexes and ranges from 10% to 40% 13 14 15 16 . The polyps are often associated with chronic and recurrent inflammation, resulting in subsequent attempts of the mucosa to heal and regenerate. Nonetheless, their prevalence does not increase with longer disease duration. Jalan and colleagues reported that post-inflammatory polyps were evident in one-third of UC patients during the first 5 months of the disease 17 *.* De Dombal and colleagues showed that about 10% of 204 patients included in the study had post-inflammatory polyps at time of the first flare-up 14 .

Morphology of post-inflammatory polyps is heterogeneous. They are usually small and, when greater than 1.5 cm in size, are called “giant pseudopolyps” *.* A particular form of post-inflammatory polyp is the filiform polyp, which appears slender, fingerlike, and looks like a polyp stalk without a head and often with branching. Another distinct form is the bridged post-inflammatory polyp, representing a mucosal bridge among polyps. In 1999, Rubin et al. 18 and more recently Sussman et al. 19 described macroscopic features of post-inflammatory polyps characterized by a smooth surface, with exudates, sharp borders, and usually multiple. However, these descriptors have not been subsequently validated by larger cohorts.

Evidence emerging from large cohort or case-control studies indicates that IBD patients with post-inflammatory polyps have an increased risk of CRN 13 14 15 20 21 22 . More recent large cohort studies and meta-analyses have challenged this concept, indicating that post-inflammatory polyps per se do not independently increase risk of advanced neoplasia once disease extent and inflammatory activity are taken into account.. In a retrospective cohort study, de Jong et al. showed that post-inflammatory polyps were associated with extensive disease, inflammation, and higher rates of colectomy, but cumulative incidence of advanced neoplasia was comparable between patients with or without post-inflammatory polyps 23 . Another retrospective study compared two populations based on presence or absence of post-inflammatory polyps and histologic IBD activity assessed by the Nancy index. This study showed that histological activity of the disease and not presence of post-inflammatory polyps increased risk of CRN 24 . In a recent meta-analysis of eight studies, it was reported that patients with post-inflammatory polyps were at higher risk of CNR, but this association was not confirmed in the pooled hazard ratio analyses 25 . The reason for such a discrepancy is unknown, although it could reflect differences in study design and heterogeneity of the population analyzed. Another possibility is that IBD-associated post-inflammatory polyps, even in the same patient, can exhibit differences in genetic and molecular pathways, some of which could be linked to the development of CNR.

## Endoscopic assessment and management of post-inflammatory polyps

**Fig. 1:**
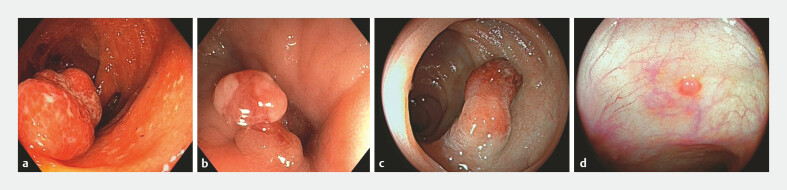
Representative endoscopic images of post-inflammatory polyps detected in the colon of patients with long-standing colitis, in **a, b, c** white light and **d** linked color imaging (LCI).

Surveillance colonoscopy is crucial for detecting dysplasia in patients with long-standing colonic IBD. However, in mucosal areas characterized by presence of multiple polypoid lesions, it is mandatory to endoscopically differentiate post-inflammatory polyps from neoplastic polyps. Chronic inflammation can profoundly distort mucosal architecture and pit pattern morphology, reducing reliability of conventional pit pattern-based classifications in IBD and contributing to both false-positive and false-negative assessments 26 . Although newer technologies, such as narrow-band imaging (NBI) and chromoendoscopy (CE), have enhanced detection of polypoid lesions, their ability to differentiate inflammatory polyps from neoplastic lesions remains unproven. Endoscopic images of post-inflammatory polyps detected in the colon of patients with long-standing colitis are shown in Fig. 1 . Currently, the SCENIC consensus recommends dye CE with targeted biopsies as the standard for IBD patients 27 . However, data on diagnosing and managing post-inflammatory polyps are limited. The Kudo classification, used with CE and magnification, helps differentiate colonic polyps in the general population. However, no systematic studies have been conducted on non-neoplastic lesions in UC, where inflammation can distort pit patterns regardless of dysplasia, and conflicting results have been produced about usefulness of these technologies in evaluation of polypoid lesions in IBD patients 19 28 29 30 31 32 . Use of Fuji Intelligent Color Enhancement (FICE) with the Kudo classification was evaluated by Cassinotti in a prospective study. The authors showed that Kudo classification is not accurate enough to differentiate neoplastic from non-neoplastic lesions because false positivity was seen in more than one-quarter of lesions 33 .

Histologic sampling or removal of polypoid lesions in IBD patients is recommended only for “suspicious” lesions. Features of underlying malignancy include uneven redness, nodularity, villous texture, slight elevation or depression, friability, obscured vascular pattern, ulcerated or velvety surface, disruption of innominate lines, and inability to lift with submucosal injection. In cases in which there is doubt about morphology of post-inflammatory polyps, or in the presence of multiple polyps and/or large size compromising endoscopic surveillance, either multiple biopsies, even in repeated examinations, or endoscopic/surgical removal of the lesion is recommended. A prophylactic colectomy should be considered when multiple post-inflammatory polyps and/or uncontrolled inflammation make it difficult to identify suspected lesions.

Polypectomy, endoscopic mucosal resection (EMR), and endoscopic submucosal resection (ESD) should be considered for suspicious or symptomatic polyps. Although European and American guidelines suggest sampling of biopsies from the area immediately adjacent to the resected polyp, evidence supporting this practice is weak, probably reflecting the fact that improved endoscopic technologies may have eliminated the need to biopsy seemingly normal tissue to detect hidden dysplasia 34 35 . Supplementary Table 1 provides a suggested management strategy for pseudopolyp-like lesions based on their endoscopic appearance.

## Frequency and predictors of dysplasia in post-inflammatory polyps

Currently, risk of pseudopolyp degeneration is not widely recognized, even though there is evidence that neoplastic transformation can occur in some circumstances. Goldgraber et al reported a case series of several forms of post-inflammatory polyps, with some of them showing premalignant changes regardless of their size 36 . Through analysis of DNA samples extracted from 30 different post-inflammatory polyps, Jawad et al. documented some protumorigenic mutations in some subsets of lesions 37 . Chromosomal changes in IBD-associated post-inflammatory polyps were also described by Lozyns’ka et al. 38 . In line with such data, our single-center experience showed that a subset of pseudopolyp-like lesions resected or biopsied during surveillance harbored dysplastic foci. These findings should be interpreted with caution because they are hypothesis-generating rather than evidence of a causal role of post-inflammatory polyps in carcinogenesis. In this study, multivariate analysis showed that patient age and the size and right colonic location of the post-inflammatory polyps were independent predictors of dysplasia, whereas previous exposure to immunosuppressors/biologics and left colonic location of lesions were inversely correlated with dysplasia 39 . Importantly, recent prospective data further support the concept that endoscopically suspected post-inflammatory polyps represent a heterogeneous group of lesions. In a single-center prospective study, dysplasia was identified in approximately 12% of endoscopically resected pseudopolypoid lesions, with more than half ultimately classified as conventional adenomas and a smaller proportion as IBD-associated dysplasia, highlighting a significant rate of endoscopic misclassification 40 . These findings highlight a significant degree of endoscopic misclassification, reflecting the difficulty of reliably distinguishing inflammatory from neoplastic lesions in chronically inflamed mucosa based on macroscopic appearance alone. In a recent review of different histologic features associated with carcinoma in IBD, Gui and colleagues underscored that colitis-associated dysplasia exhibited a mixed/intermingled appearance with inflammatory polyps and/or granulation tissue, revealing a histologic overlap and confirming the difficulty in differentiating the lesions 41 .

These results challenge, to a greater extent, the notion that post-inflammatory polyps are uniformly harmless and suggest that some of them may represent or conceal dysplastic changes.

## Potential role of AI in endoscopic assessment of post-inflammatory polyps

AI has emerged as a promising adjunct to enhance dysplasia and CRC detection during surveillance endoscopy in IBD, where subtle and flat lesions may easily escape human eye detection. Several AI-assisted systems using convolutional neural networks have demonstrated high accuracy in identifying colitis-associated neoplasia with encouraging results in experimental and early clinical settings. In a pilot study, Yamamoto et al. developed an AI system capable of classifying IBD-associated lesions into high-grade vs. low-grade/sporadic groups, outperforming both expert and non-expert endoscopists (diagnostic accuracy: AI 79.0%, experts 75.8%, non-experts 77.8%) 42 . Similarly, Misawa et al. applied the EndoBRAIN-EYE system to IBD patients and successfully highlighted flat dysplastic lesions otherwise difficult to detect 43 . More recently, Vinsard et al. evaluated a dedicated IBD computer-aided detection model trained on over 3400 IBD surveillance colonoscopies, showing excellent performance for dysplasia detection in white-light endoscopy (sensitivity 95.1%, accuracy 96.8%). However, its performance dropped significantly in chromoendoscopy settings (accuracy 77.8%), suggesting limitations in complex mucosal landscapes.

Despite these promising advances, no AI model has yet been validated to reliably differentiate post-inflammatory polyps from dysplastic lesions in IBD. Current AI systems are primarily designed for lesion detection rather than lesion characterization and are not specifically trained to recognize inflammatory post-inflammatory polyps. This limitation is particularly relevant, given that post-inflammatory polyps are common in long-standing IBD and may both obscure and mimic dysplastic lesions. Moreover, advanced imaging criteria, such as pit pattern or surface texture, often fail to distinguish post-inflammatory polyps in IBD due to inflammation-related distortion, and these challenges extend to AI-based image interpretation.

To bridge this gap, future developments should include integration of large, multicenter, high-quality endoscopic imaging datasets capturing the full spectrum of inflammatory and neoplastic lesions, including post-inflammatory polyps, inflammatory lesions, and dysplasia-associated lesions; systematic correlation with complete histological assessment of resected lesions; and robust external validation across independent cohorts and different endoscopic platforms. Such training would enable development of IBD-specific AI tools capable not only of detecting lesions but also of accurately characterizing their neoplastic potential, thereby improving both precision and safety of surveillance strategies in complex IBD cases.

## Conclusions

The findings described in this article indicate that management of post-inflammatory polyps in patients with longstanding and extensive IBD remains a major clinical challenge because we do not yet know exactly whether and which lesions are linked to development of CRC in such patients. Pioneering large cohort and case-control studies showed that presence of post-inflammatory polyps increased risk of colon carcinogenesis in IBD, even though no molecular explanation was provided to justify the link between post-inflammatory polyps and IBD-related CRC development 13 15 20 21 22 . It was suggested that this association simply could reflect the more intense activity of mucosal inflammation, because post-inflammatory polyps are considered a marker of chronically active colitis. Alternatively, presence of post-inflammatory polyps, especially when they are numerous and alter colonic normal morphology, might obscure otherwise visible and resectable dysplastic lesions. However, additional studies and a recent meta-analysis did not confirm the association between post-inflammatory polyps and CRC in IBD, probably reflecting differences in methodologies adopted in such studies 24 25 .

Another and less explored possibility is that some subsets of pseudopolyp-like lesions can eventually transform into CRN. This hypothesis is supported by the demonstration that pro-tumorigenic mutations and chromosomal anomalies can be detected in some post-inflammatory polyps, and one-fourth of them evident during surveillance colonoscopy bear dysplastic foci 37 39 40 . These findings further challenge the historical view of post-inflammatory polyps as purely benign post-inflammatory sequelae and support emerging evidence underscoring the need to better understand their true clinical significance.

However, further and prospective clinical and molecular studies are needed to confirm these findings. If this hypothesis is true, then the question remains as to how we can improve endoscopic tools to allow clinicians to identify post-inflammatory polyps that bear an intrinsic risk of neoplastic progression. Recent applications of AI algorithms in identification and characterization of intestinal polyps and colorectal cancer predictions suggest that AI-based prediction models have the potential to help address the issue of CRC development in IBD patients with post-inflammatory polyps.

It is also important to emphasize that training of AI systems applied to endoscopy, which holds great potential for improving accurate recognition of post-inflammatory polyps, relies on availability of correctly identified and annotated lesions.

Although the available evidence does not support a paradigm shift in which post-inflammatory polyps are regarded as independent precursors of colorectal cancer, growing data suggest that a minority of pseudopolyp-like lesions may conceal or coexist with dysplasia, highlighting the need for improved diagnostic stratification rather than a redefinition of their biological nature. In particular, endoscopic differentiation between inflammatory and dysplastic lesions remains challenging because chronic inflammation can significantly distort mucosal architecture and pit pattern morphology, leading to irregular or disorganized. This limitation reduces reliability of conventional pit pattern-based classifications in IBD and contributes to both false-positive and false-negative assessments. In this context, multicenter studies combining careful endoscopic selection, systematic histological and molecular characterization of resected lesions, and integration of advanced imaging and AI-based tools will be essential to better identify lesions that warrant closer surveillance or intervention.

## Supplementary Material

Supplementary Material Zusatzmaterial
