# Supplementary material for: Post-inflammatory polyps and risk of dysplasia in inflammatory bowel disease: Wolves in sheep’s clothing?
Source: Endosc Int Open. 2026 Mar 16;14:a28189346. doi: 10.1055/a-2818-9346 (PMC13063304; doi:10.1055/a-2818-9346)
Supplement: Supplementary file 1 — Supplementary Material [file 10-1055-a-2818-9346_28225704.pdf]

Supplementary Table 1 Endoscopic features raising suspicion for dysplasia in pseudopolyp-like lesions and corresponding suggested management strategies.

	Features raising suspicion for dysplasia	Suggested management
**Vascular pattern**	Focal vascular irregularity or abnormal vessels	Targeted biopsy or resection
**Pit pattern**	Heterogeneous, irregular, or disrupted pit pattern	Prefer resection
**Dye-chromoendoscopy**	Focal dye exclusion or irregular staining	Targeted biopsy or resection
**Ulceration**	Absence of ulceration in polypoid lesions	Biopsy or follow-up
**Multiplicity**	Solitary or dominant lesion among multiple pseudopolyps	Targeted biopy or resection

Endosc Int Open | DOI 10.1055/a-2818-9346 | © The Author(s).
